# Disassembly of Shank and Homer Synaptic Clusters Is Driven by Soluble β-Amyloid_1-40_ through Divergent NMDAR-Dependent Signalling Pathways

**DOI:** 10.1371/journal.pone.0006011

**Published:** 2009-06-23

**Authors:** Francesco Roselli, Peter Hutzler, Yvonne Wegerich, Paolo Livrea, Osborne F. X. Almeida

**Affiliations:** 1 Max-Planck Institute of Psychiatry, Munich, Germany; 2 Department of Neurological and Psychiatric Sciences, University of Bari, Bari, Italy; 3 Institute of Pathology, Helmholtz Center Munich, Neuherberg, Germany; Tokyo Medical and Dental University, Japan

## Abstract

Disruption of the postsynaptic density (PSD), a network of scaffold proteins located in dendritic spines, is thought to be responsible for synaptic dysfunction and loss in early-stage Alzheimer's disease (AD). Extending our previous demonstration that derangement of the PSD by soluble amyloid-β (Aβ) involves proteasomal degradation of PSD-95, a protein important for ionotropic glutamate receptor trafficking, we now show that Aβ also disrupts two other scaffold proteins, Homer1b and Shank1, that couple PSD-95 with ionotropic and metabotropic glutamate receptors. Treatment of fronto-cortical neurons with soluble Aβ results in rapid (within 1 h) and significant thinning of the PSD, decreased synaptic levels of Homer1b and Shank1, and reduced synaptic mGluR1 levels. We show that *de novo* protein synthesis is required for the declustering effects of Aβ on Homer1b (but not Shank1) and that, in contrast to PSD-95, Aβ-induced Homer1b and Shank1 cluster disassembly does not depend on proteasome activity. The regulation of Homer1b and Shank1 by Aβ diverges in two other respects: i) whereas the activity of *both* NMDAR *and* VDCC is required for Aβ-induced declustering of Homer1b, Aβ-induced declustering of Shank1 only requires NMDAR activity; and ii) whereas the effects of Aβ on Homer1b involve engagement of the PI-3K pathway and calcineurin phosphatase (PP2B) activity, those on Shank1 involve activation of the ERK pathway. In summary, soluble Aβ recruits discrete signalling pathways to rapidly reduce the synaptic localization of major components of the PSD and to regulate the availability of mGluR1 in the synapse.

## Introduction

Loss of synaptic structure and function is thought to mark early stages of Alzheimer's disease [1]. Soluble amyloid-β (Aβ) peptides dramatically derange synaptic plasticity, dendritic spine number and motility, and pre- and postsynaptic composition without overt neuronal loss [2]–[5]. Notably, cognitive disturbances are significantly correlated with levels of soluble Aβ oligomers in both AD patients and transgenic mouse models [3], [6]–[8], and Aβ oligomers produce transient cognitive deficits when injected into the brains of healthy animals [2].

The morphological and functional integrity of synapses, and in particular of post-synaptic sites, requires the proper organization of the post-synaptic density (PSD), a network of scaffold proteins and enzymes located in dendritic spines [9]. The PSD is composed of a large number of interacting scaffold proteins, including a PSD95/NMDAR module and a Homer1b/mGlurI complex, both of which interact with Shank family members [10]; PSD-95, Homer1b and Shank are all involved in the regulation of dendritic spine formation [11]–[13]. Homer 1b is localized at the periphery of the PSD [14], [15]; its N-terminal EVH1 domain binds and modulates metabotropic glutamate receptor (mGluR) trafficking and signalling [16], [17]. The EVH1 domain of Homer also interacts with Shank family proteins [14], [18]. Shank1 is located on the cytoplasmic side of the PSD [15] and forms a core element of the framework of the PSD [19]. Among others, Shank1 has a PDZ domain that is essential for its interaction with the GKAP/PSD-95/NMDAR complex, and a proline-rich domain which serves as a docking site for Homer1b/mGluR I and for several actin cytoskeleton-related proteins such as cortactin [10], [20]; these features allow Shank1 to physically bridge the actin cytoskeleton with ionotropic and metabotropic glutamate receptors.

Evidence supporting the hypothesis that disassembly of the PSD may be an early event in Aβ-induced synaptic dysfunction includes the observation that Aβ oligomers induce the proteasomal degradation of a major post-synaptic density protein (PSD-95), followed by endocytosis of NMDA and AMPA receptors [21], [22]. However, the full extent of the PSD remodelling by Aβ oligomers has not yet been explored. Given the relative abundance and important functions of Homer1b and Shank [12], [23], this study analyzed the dynamics and mechanisms of regulation of these post-synaptic proteins by Aβ. The results demonstrate that, in addition to inducing degradation of PSD-95 [21], [22], soluble Aβ causes rapid changes in PSD architecture by depleting the synaptic pool of Homer1b and Shank1 clusters, culminating in reduced synaptic levels of mGluR1 in cortical neurons. Further, it is shown that distinct signalling pathways mediate the actions of soluble Aβ on Homer1b and Shank levels.

## Materials and Methods

### Compounds

Aβ_1–40_ (American Peptides, Sunnyvale, CA) was prepared as previously described [21] to yield predominantly low N-oligomers (mainly monomeric to tetrameric) [24]–[26]. While the preparation may have contained a few protofibrils, it is pertinent to note that longer incubation times and higher concentrations of the peptide (>10 µM) are required for fibrillogenesis [27], [28]. Nifedipine, (±)-verapamil, roscovitine, NiCl_2_, cycloheximide, cyclosporin and FK506 were purchased from Sigma Chemicals (Deisenhofen, Germany); NMDA, Bay-K4688, SL0101-1, MK801, UO126, PD98059, API-2, wortmannin, cantharidine were from Tocris (Bristol, UK); and sodium orthovanadate, rapamycin, TDZT, SU6656 and MG132 were from Calbiochem (La Jolla, CA).

### Primary cell culture

Trypsin-dissociated primary cell cultures were prepared from frontal cortical tissue from 4-day-old (P4) Wistar rats (Charles River, Sulzfeld, Germany), using a previously described method [22], [29]. Cells were plated onto gelatin/PDL-coated glass coverslips (450,000 cells/mm^2^). Experiments were started after 10–13 days *in vitro* (DIV 10–13). When required, cells were starved by culturing them in Neurobasal A medium/1 mM glutamine (Invitrogen, Karlsruhe, Germany) for 2 h before treatments.

### Electron microscopy

Neurons were grown on Permanox slides (LabTek, Nalgene, Naperville, IL) at 600 cells/mm^2^ and used after DIV 10–13. After treatment cells were washed in PBS and fixed in 3% PFA/3% glutaraldehyde/0.1 M cacodylate buffer (Electron Microscopy Sciences, Hatfield, PA) for 20 min at 25°C, before washing and post-fixation in 2% osmium tetroxide. After dehydration, specimens were embedded in Epon resin (Electron Microscopy Sciences), cut (70 nm) and collected on copper grids, and stained with 0.5% uranyl acetate and 3% lead citrate. Grids were examined on a EM10 Zeiss electron microscope (acceleration voltage: 60 kV). Images were acquired at 50,000× original magnification and at resolution of 1376×1036 pixels. Only artifact-free synapses, with clearly identifiable presynaptic terminals, synaptic clefts, postsynaptic membranes and PSD were selected [30]; our analysis focused on glutamatergic synapses, identified by the presence of PSD located in dendritic spines. Thickness and length of the PSD were measured using ImageJ software [31]. Images were acquired from 6 independent specimens (N = 6); 15–20 synapses were evaluated in each block (total number of spines: control, n = 112; treated, n = 94).

### Western blotting

Cells were lysed, electrophoresed (6% or 12% acrylamide gels) and blotted on nitrocellulose membranes as previously described [21]. Membranes were incubated with one of the following primary antibodies: anti-Homer1b/c (1∶2,000 Santa Cruz Biotechnology, Heidelberg, Germany or 1∶3000, Synaptic Systems, Goettingen, Germany), anti-synapsin I (1∶2,000; Chemicon, Chandlers Ford, UK), and either anti-actin (1∶10,000; Chemicon) or anti-β-tubulin (1∶2,000; Oncogene Science, Schwalbach, Germany) before staining with appropriate horseradish peroxidase-IgG conjugates (GE Health, Freiburg, Germany). Immunoreactivity was revealed by enhanced chemoluminescence (GE Health) and optical densities were measured, after checking for linearity of signal, using TINA 3.0 bioimaging software (Raytest, Straubenhardt, Germany). All values were normalized and expressed as percentages of control; in pharmacological experiments, percentages of responses to Aβ treatment vs. control treatment. Each set of numerical data shown was obtained from 3 to 5 independent sets of experiments, with 3 replicates in each run.

### Immunostaining and confocal imaging

Cells were fixed and blocked as previously described [21] before overnight incubation (4°C) with anti-Homer1b/c (1∶500; Santa Cruz), anti-Shank1 (1∶200) or anti pan-Shank (1∶400) (both kind gifts of M. Sheng) and subsequent incubation with Alexa-488 conjugated goat anti-rabbit IgG (1∶500; Molecular Probes, Eugene, OR). Double-staining with synaptophysin was performed using rabbit anti-synaptophysin (1∶400; Sigma) and Alexa-543 conjugated goat anti-mouse IgG (1∶500; Molecular Probes). For mGluR1, p-PKB, p-Erk and p-RSK immunostaining, cells were fixed in 4% *p*-formaldehyde (pH 7.4; 5 min; RT), before washing, overnight (4°C) incubation with primary antibody (1∶250 anti-mGluR1, Sigma; 1∶200 anti-p-PKB (Thr308), 1∶250 anti-p-ERK1/2 (Thr202/Tyr204), and 1∶100 anti p-RSK 1/2/3 (Ser-380), all from Cell Signalling/NEB, Frankfurt am Main, Germany) and Alexa-488 conjugated goat anti-rabbit IgG (1∶500). These cells were subsequently stained for synaptophysin as described above. Surface mGluR1 was detected after fixing cells in 4% PFA, as above but with omission of the permeabilization step. Cells were incubated with anti-mGluR1 (1∶100, Alomone laboratories, Jerusalem, Israel) for 1 h at 25°C and with Alexa-488 conjugated goat anti-rabbit IgG (1∶500). In all cases, coverslips were mounted with Vectashield (Vector Labs, Burlinghame, CA).

Optical section images and stacks of images from fluorescence-labelled cells were obtained using a confocal laser scanning microscope (LSM 510; Carl Zeiss, Jena, Germany) equipped with a plan-apochromat 63×/1.2 water lens, and where 1 pixel corresponded to 0.1 µm^2^. Homer1b, Shank1, synaptophysin and mGluR1 immunostaining was monitored in 275–375 puncta within 8–12 randomly chosen dendrites from 6–8 neurons (triplicate specimens). Dendrites showing less than 25 synapses were not considered in the analysis. Image analysis was carried out using ImageJ 1.37c software (NIH, Bethesda, MD). To evaluate cluster size, single channel images were extracted, collapsed along the z-axis using the maximum intensity algorithm, and thresholded at the arbitrary values of 80 (Homer1b, Shank1 and synaptophysin), 230 (surface mGluR1), or 135 (total mGluR1). Homer1b, Shank1 and mGluR1 clusters that were juxtaposed to synaptophysin puncta in overlay images were manually selected by an investigator who was blind to the treatment; surface areas (pixels) were measured with ImageJ software and logged into an Excel file. Clusters separated by 1 pixel were considered to represent individual clusters. To reduce noise effects, only clusters ≥3 pixels were included in the analysis. This criterion possibly introduced a bias toward smaller differences in treated *vs.* untreated groups in cases where treatment caused a shrinkage of a substantial proportion of clusters to <3 pixels; however, since this set-point would rather lead to an overestimation of average cluster size in the treated groups, the statistical significance of detected differences would not be undermined. The above cluster size analysis was complemented by independent evaluation of the images by a second investigator (also blind to treatments) who ranked images according to cluster size; in all cases, there was a 100% match between these latter qualitative evaluations and the quantitative analysis. Colocalization was calculated as the number of puncta displaying positive synaptophysin and Homer1b immunoreactivity/total number of synaptophysin-positive puncta. Only puncta ≧3 pixels were considered to be clusters and included in the analysis. For each condition, 8–12 neurons from 3–4 experiments were considered (N = 8–12), and at least 50 synapses on each neuron were evaluated (total puncta n = 400–600).

The fluorescence intensity of p-PKB, p-ERK and p-RSK was calculated using single optical sections 1 µm thick (full-width, half-maximum); regions of interest were traced in synaptophysin-positive cell bodies. For each condition, 30 to 50 neurons from three replicates were evaluated. Average fluorescence intensities were logged into Excel files, after subtraction of the local background value for each spot.

*Statistical analysis*: All data are depicted as means±S.D. from 3–5 independent experiments. Data shown represent the number of neurons (N) analyzed. Data were tested for statistical significance using ANOVA and appropriate *post-hoc* tests, with p<0.05 being set as the minimum level of significance.

## Results

### Aβ induces ultrastructural changes in the PSD

**Figure 1:**
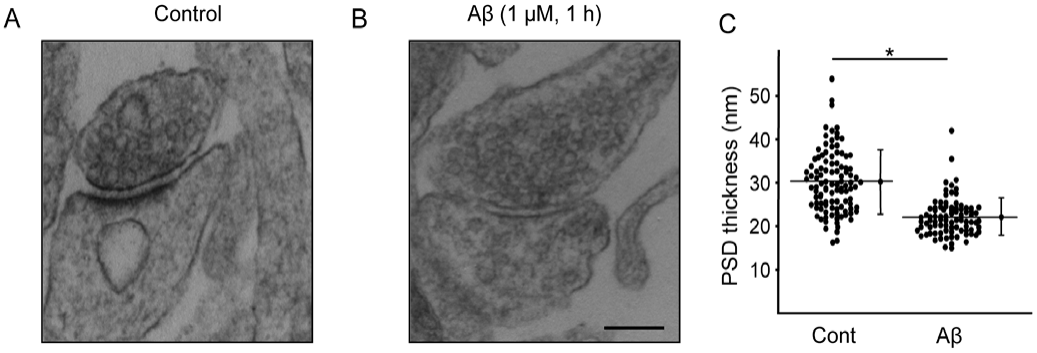
Ultrastructural effects of Aβ on the post-synaptic density (PSD). Rat fronto-cortical neurons in culture were treated with vehicle or Aβ (1 µM, 1 h) before being fixed, embedded and sectioned at 70 nm for transmission electron microscopy (TEM). (A, B) Aβ decreases the thickness of the post-synaptic density. Representative TEM images show a significant decrease in PSD thickness after Aβ treatment. Scale bars represent 500 nm. (C) After Aβ treatment, PSD thickness was reduced to 22.12±4.2 nm vs. 30.22±7.8 nm in vehicle-treated neurons (p<0.001). Images were acquired from 6 independent specimens (N = 6), and 15–20 synapses in each block were evaluated (total number of spines: control, n = 112; treated, n = 94). Data shown are mean±SD.

The PSD, a prominent feature of excitatory post-synaptic sites, can be visualized by transmission electron microscopy as an electron-dense thickening of the post-synaptic membrane. Under control conditions, the PSD of asymmetric synapses of cultured neurons was found to display morphological features, including length (302±92.1 nm) and thickness (30.22±7.8 nm), that are typical of such synapses in adult brain (Fig. 1A) [cf. 15]. Treatment of cultures with 1 µM soluble Aβ_1–40_ (hereinafter referred to as Aβ) led to a rapid and significant thinning of the PSD (22.12±4.2 nm, n = 94, p<0.001; Fig. 1A, B and C) and a leftward shift in the cumulative frequency distribution of PSD thickness (not shown); a shorter exposure to Aβ (30 min) did not significantly affect PSD thickness (29.8±7.14 nm).

### Reduced Homer1b and Shank clustering in synapses after Aβ treatment

Since Homer1b and Shank1 are major structural constituents of the PSD, the influence of Aβ on the synaptic pools of these proteins was studied by monitoring the size and distribution of Homer1b and Shank1 clusters at synaptic sites. In untreated fronto-cortical neurons, punctate Homer1b staining was clearly evident along the dendrites whereas only faint staining was detectable in dendritic shafts; the majority of Homer1b clusters (83.3±6.5%) were juxtaposed to synaptophysin-positive puncta, indicating synaptic localization of Homer1b in mature neurons. Punctate Shank1 staining was observed in close apposition to 84.4±3.9% of synaptophysin-immunoreactive puncta. Homer and Shank cluster sizes were dose-dependently influenced by Aβ at doses ranging between 10 nM and 1 µM (Fig. S1A and B); more detailed analyses were performed with Aβ at a dose of 1 µM.

**Figure 2:**
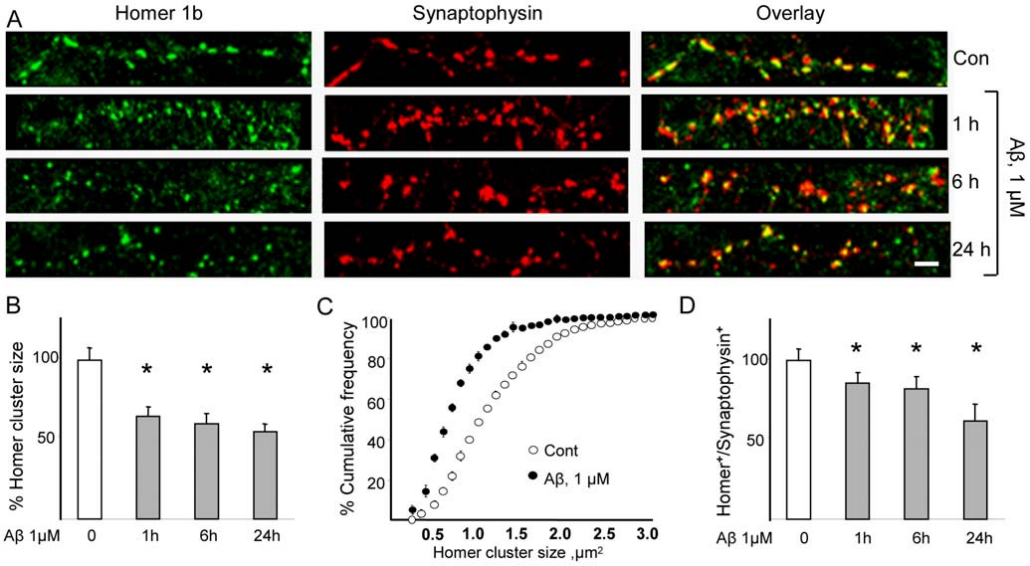
Aβ induces the dispersal of Homer1b synaptic clusters. Fronto-cortical neurons in culture were treated with vehicle or Aβ (1 µM) for 1, 6 or 24 h before immunostaining. (A, B) Homer1b cluster size is significantly decreased after 1 h treatment with Aβ (64.9±5.9% vs. 100±7% in control; p<0.05); a similar decrease is observed after 6 and 24 h of exposure to Aβ (59.7±6.7% and 55±4.9% of control, respectively; p<0.05). (C) Aβ alters Homer1b cluster size distribution. Homer1b cluster size distribution was analyzed after 1 h of treatment with Aβ or vehicle. The cumulative frequency plot shows a homogeneous leftward shift in the frequency curve after Aβ treatment. (D) Aβ decreases the number of synaptophysin-labelled synapses that co-localize Homer1b. The ratio of Homer1b to synaptophysin immunopositive puncta was decreased after 1 h treatment with Aβ (85.6±6.8% vs. control; p<0.05), and further decreases were observed after treatment for 6 and 24 h (82.3±7.8% and 61.9±10.4% of control, respectively; p<0.05 in both cases). For each condition 8–12 neurons from 3–4 replicate experiments were considered (N = 8–12), and at least 50 synapses on each neuron were evaluated (on average, n = 500 puncta/condition). Scale bars represent 5 µm.

Exposure of neurons to Aβ (1 µM, 1 h) resulted in a significant decrease in Homer1b cluster size (64.9±5.9% *vs.* 100±7% in controls, p<0.05; Fig. 2A and 2B) and a marked leftward shift in cluster size distribution (Fig. 2C). Longer exposures to Aβ (6 and 24 h) did not produce further changes in Homer1b cluster size (Fig. 2A and B). Nevertheless, the number of synapses showing undetectable levels of Homer1b increased with time of exposure to Aβ; thus, the abundance of apposed Homer1b/synaptophysin-immunoreactive puncta decreased to 85.6±6.8% (1 h), 82.3±7.8% (6 h) and 61.9±10.4% (24 h) of baseline (100%) value after exposure to Aβ (Fig. 2D).

**Figure 3:**
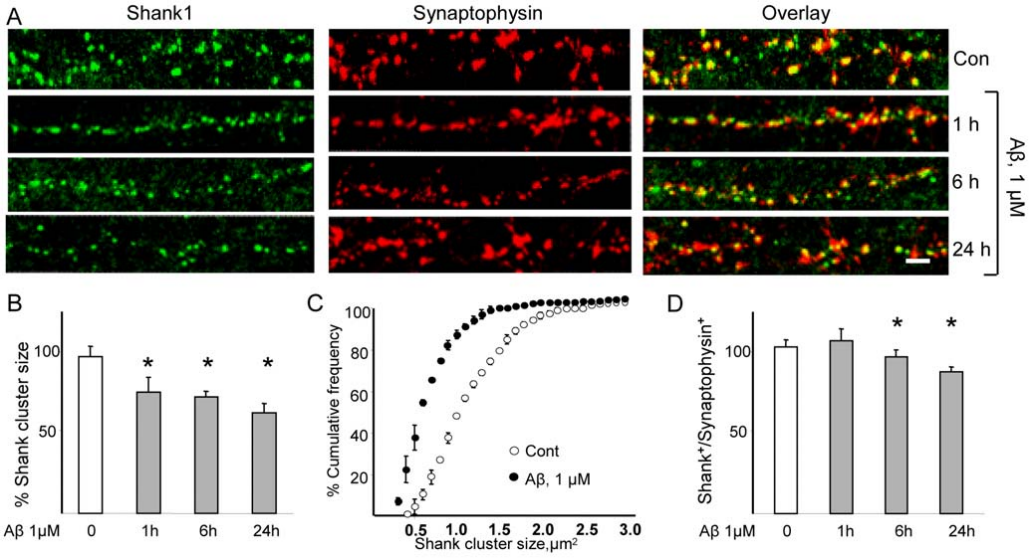
Aβ induces the dispersal of Shank1 synaptic clusters. Fronto-cortical neurons in culture were treated with vehicle or Aβ (1 µM) for 1, 6 or 24 h before immunostaining. (A, B) Quantification of Shank1 clusters revealed a significant decrease in average size within 1 h of treatment with Aβ (77.6±9.4% vs. control; p<0.05); a similar decrease was seen after 6 and 24 h (74.5±3.6% and 64.4±5.8% of control, respectively; p<0.05). (C) Analysis of Shank1 cluster size distribution after 1 h of treatment with Aβ (plotted according to cumulative frequency) revealed that Aβ leads to a homogeneous leftward shift in the frequency curve. (D) Aβ decreases the number of Shank1-immunopositive synapses; synaptic sites were marked with anti-synaptophysin. The ratio of Shank1 to synaptophysin immunopositive puncta was not decreased (compared to control) after 1 h Aβ treatment (103.7±7.4; p = 0.075), but was significantly reduced after exposure to Aβ for 6 and 24 h (94.2±4.5% and 84.9±3.2%, respectively; p<0.05). For each condition 8–12 neurons from 3–4 replicate experiments were considered (N = 8–12), and at least 50 synapses on each neuron were evaluated (on average, n = 500 puncta/condition). Scale bars represent 5 µm.

Exposure of cells to Aβ for 1 h resulted in a significant reduction of Shank1 cluster size (77.6±9.4% of controls, p<0.05; Fig. 3A and B) and a leftward shift in the Shank1 cluster size distribution curve (Fig. 3C). Extended exposure to Aβ resulted in further reductions in Shank1 cluster size (74.5±3.6% after 6 h; 64.4±5.8% after 24 h; Fig. 2E and F). Interestingly, the rate at which the ratio of Shank1/synaptophysin apposition shifted after Aβ treatment was slower than that observed for Homer1b/synaptophysin, with the first significant change occurring after 6 h (94.2±4.5% of control, p<0.05) and a further reduction after 24 h (84.9±3.2% of control, p<0.05) of Aβ application (Fig. 3D).

### Aβ-induced decreases in synaptic Homer1b and Shank1 levels occur in a proteasome-independent manner

**Figure 4:**
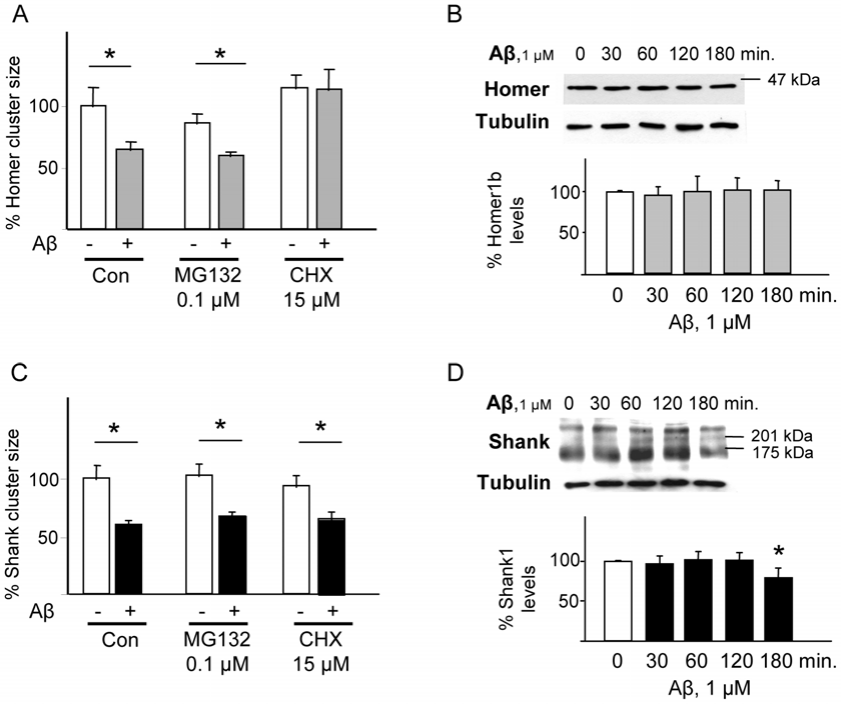
Aβ-induced dispersal of Homer1b and Shank1 clusters does not depend upon protein degradation. Protein synthesis is required for Aβ-induced Homer1b clusters dispersal. Neurons were pretreated with the proteasome inhibitor MG132 (0.1 µM) or with the protein synthesis inhibitor cycloheximide (CHX, 15 µM) before adding Aβ (1 µM), before immunostaining for synaptophysin and either Homer1b or Shank1. (A) Proteasome inhibition did not block the Aβ effects (69.1±4.2%, cf. Aβ+MG132 and MG alone, p<0.05), while blockade of protein synthesis effectively prevented the dispersal of Homer1b clusters (103.9±10%, cf. CHX+Aβ and CHX alone). For each condition, 8–12 neurons from 3–4 replicate experiments were considered (N = 8–12), and at least 50 synapses on each neuron were evaluated (average, n = 500 puncta/condition). (B) Aβ did not reduce whole cell levels of Homer1b levels after 30, 60, 120 or 180 min. (C) Neither proteasomal activity nor *de novo* protein synthesis were found to be necessary for Aβ-induced dispersal of Shank1 clusters. (D) Exposure to Aβ for up to 120 min. did not lead to down-regulation of Shank1 levels in whole cell protein extracts; however, a significant decrease in Shank1 was observed after 180 min of exposure to Aβ (80.7±9.7% of baseline; p<0.05). Since Shank1 displays multiple isoforms, the quantification shown refers to the most abundant (170 kDa) band.

Synaptic protein abundance and turnover are determined by de novo protein synthesis and proteasome activity [32]; the latter was previously demonstrated to be essential for Aβ-induced degradation of PSD-95 [21]. Here, we found that pre-treatment of neurons with the proteasome inhibitor MG132 (0.1 µM) did not prevent Aβ-induced reductions in synaptic Homer1b (Fig. 4A) and Shank1 (Fig. 4C) cluster sizes. Furthermore, whole-cell lysate levels of both proteins remained constant after application of 1 µM (Fig. 4B and D) or 10 µM (not shown) of Aβ. Confirming the findings for Homer1b, antibodies directed against either the N- or C-terminal epitopes of Homer1b failed to detect truncated forms of the protein (data not shown).

In examining the importance of protein synthesis for the manifestation of the Aβ effects on synaptic levels of Homer1b and Shank1, we observed differential dependence of the regulation of these two proteins by Aβ. Specifically, co-application of the protein synthesis inhibitor cycloheximide (CHX, 15 µM) prevented Aβ-induced shrinkage of Homer1b clusters at synaptic sites (Fig. 4A), but did not interfere with the ability of Aβ to reduce the size of Shank1 clusters (Fig. 3C).

### Discrete Ca^2+^ sources contribute to Aβ actions on Homer1b and Shank1

**Figure 5:**
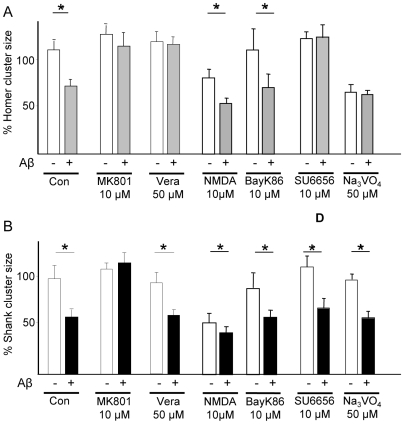
Differential requirements of NMDAR and VDCC for Aβ-induced declustering of Homer1b and Shank1. (A) Aβ-induced declustering of Homer depends on NMDAR VDCC activity and on src activity. Cells were pre-treated with either the NMDAR antagonist MK801 (10 µM) or the VDCC blocker verapamil (50 µM) before exposure to Aβ (1 µM, 1 h). Both MK801 and VDCC effectively prevented Aβ-induced Homer1b cluster dispersal (100.7±15.5%, cf. MK+Aβ and MK alone; p>0.05; 105.7±7%, cf. verapamil+Aβ and verapamil alone; p>0.05). To evaluate the sufficiency of each of these receptors for Aβ-induced Homer cluster dispersal cells were pre-treated with either the NMDAR agonist NMDA (10 µM) or the VDCC activator Bay-K8644 (10 µM) before exposure to Aβ (1 µM, 1 h). NMDA alone significantly reduced Homer (75.1±8.8%, p<0.05) and a further decrease was observed after NMDA+Aβ cotreatment (50.2±5.2% of untreated control). To assess the role of NMDAR tyrosine phosphorylation in the signalling leading to Homer cluster dispersal, cells were pre-treated with the src-family inhibitor SU6656 (10 µM), which blocked the ability of Aβ to reduce Homer1b (100.8±11.1%, cf. Aβ+SU6656 and SU6656 alone; p>0.05), or with the tyrosine-phosphatase inhibitor sodium orthovanadate (Na_3_VO_4_, 100 µM) alone, which led to decreases in Homer1b (57.7±7.2% of control; p<0.05) cluster sizes and occluded Aβ effect when Na_3_VO_4_ was applied before Aβ: (96±7.1%, cf. Na_3_VO_4_+Aβ and Na_3_VO_4_ alone; p>0 05). (B) Aβ-induced declustering of Shank depends only on NMDAR activity. In contrast to Homer1b declustering, MK801, but not verapamil, blocked the effects of Aβ on Shank1 clustering (116.6±10.4%, cf. MK801+Aβ and MK801 alone; p>0.05; 63.3±5.7%, cf. verapamil+Aβ and verapamil alone; p<0.05). Pretreatment with the NMDAR agonist NMDA (10 µM) or the VDCC activator Bay-K8644 (10 µM) before exposure to Aβ (1 µM, 1 h) reduced Shank cluster size (53.9±4.1% and 77.7±9.9% respectively; p<0.05) and a further decrease was observed after NMDA+Aβ and BayK8644+Aβ cotreatment (43.4±6.7% and 43.8±5.6% respectively, p<0.05). Pre-treatment with the src-family inhibitor SU6656 (10 µM) did not block the ability of Aβ to reduce Shank1 (90.5±12.2%, cf. SU6656+Aβ and SU6656 alone; p>0.05) clusters. Exposure to the tyrosine-phosphatase inhibitor sodium orthovanadate (Na_3_VO_4_, 100 µM) alone led to decreases in Shank1 (77.8±10.3% of control; cf. Na_3_VO_4_+Aβ and Na_3_VO_4_ alone p<0.05) cluster sizes but further decrease was observed after co-treatment with Aβ (47. ±9.8%, p<0.05). For each condition, 8–12 neurons from 3–4 replicate experiments were examined (N = 8–12); at least 50 synapses on each neuron were evaluated (on average, n = 500 puncta/condition).

Previous studies demonstrated the important role of NMDAR activity in the regulation of post-synaptic density scaffold proteins, including PSD95 [33] and Homer1b [34]. In the present study, pre-treatment of neurons with the NMDAR antagonist MK801 (10 µM) abolished the Aβ-induced decrease in synaptic levels (cluster size) of both Homer1b (Fig. 5A) and Shank1 (Fig. 5B), indicating the requirement of NMDAR for the effects of Aβ on the synaptic localization of these proteins. In contrast, the Aβ-induced changes in Homer1b and Shank1 clustering were not influenced by antagonism of AMPAR and class I/II mGluRs with NBQX and E4CPG, respectively (Supplementary Fig. S2A and S2B).

Earlier work reported the requirement of voltage-dependent calcium channel (VDCC) activity for the modulation of Homer1b clustering by glutamate [34]. Here we show that the effects of Aβ on synaptic Homer1b levels are abolished after pre-treatment with the L-type VDCC blockers, verapamil (50 µM; Fig. 5A) and nifedipine (40 µM, Fig. S3), but not with NiCl_2_, a T-type calcium channel blocker (Supplementary Fig. S3). The actions of Aβ on Shank1 levels were found to be verapamil-insensitive (Fig. 5B). Specific NMDAR and VDCC agonists were next employed to investigate the individual and combined roles of these signalling pathways in the mediation of the Aβ effects. Treatment with NMDA alone resulted in the disassembly of Homer and Shank clusters (p<0.05; Fig. 4C and 4D), an effect that was accentuated in the presence of Aβ (p<0.05; Fig. 5A and B). VDCC activation with Bay-K8644 (10 µM) did not affect Homer or Shank cluster sizes under any condition (Fig. 5A and B).

Having established a crucial role for NMDAR in Aβ-induced remodeling of the PSD, as well as the additional requirement of VDCC in the manifestation of Aβ effects on synaptic Homer1b levels, we next examined the involvement of NMDAR in greater detail. Focusing on the NR2B subunit of this receptor, we determined the importance of tyrosine phosphorylation of the C-terminal tail of this subunit. The NR2B subunit is the major tyrosine-phosphorylated protein in the PSD and its phosphorylation by src family kinases at multiple sites results in a docking site for various signalling molecules and enzymes [35]. In this study, pre-treatment of neurons with the src family tyrosine kinase inhibitor SU6656 (10 µM) significantly blocked the ability of Aβ to induce declustering of Homer1b (p<0.05, Fig. 5A), but not of Shank1 (Fig. 5B). Consistently, the tyrosine-phosphatase inhibitor, sodium orthovanadate (Na_3_VO_4_, 100 µM) mimicked the effects of Aβ on Homer clusters (p<0.05, Fig. 5A), while not influencing Shank clusters (Fig. 5B); pretreatment with Na_3_VO_4_ occluded the effects of Aβ.

### Homer1b and Shank1 are regulated by Aβ through divergent signalling pathways

**Figure 6:**
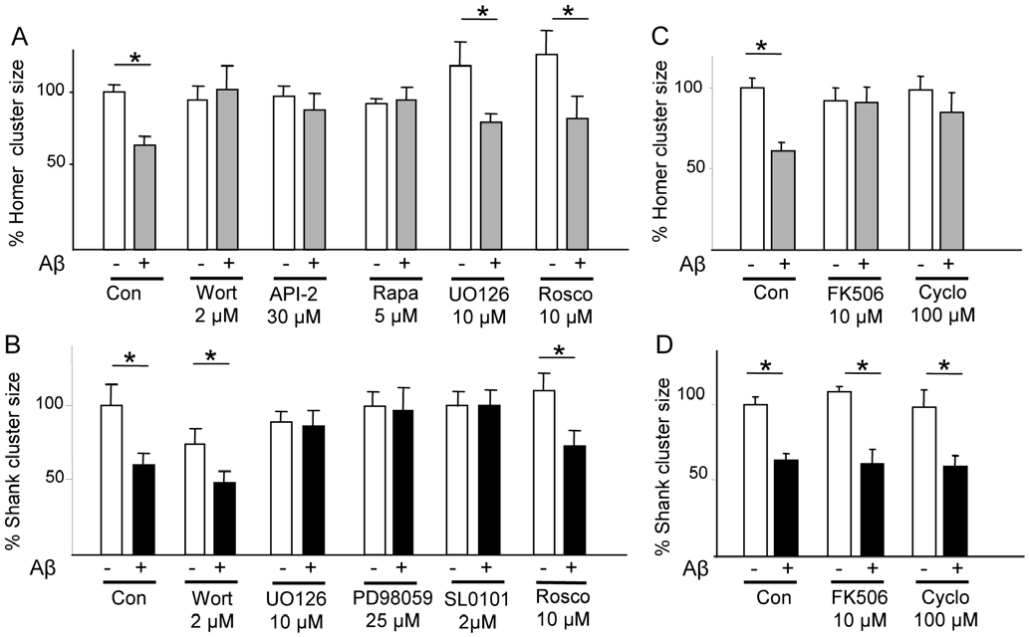
Divergent signalling pathways mediate the effects of Aβ on Homer1b and Shank1 cluster dispersal. Cultured cortical neurons were pre-treated for 45 min with the indicated pharmacological inhibitors before being treated for 1 h with Aβ (1 µM). Inhibitors were present in the medium during Aβ treatment. (A) PI-3K/mTOR pathway is required for Homer1b declustering. Pre-treatment with wortmannin (Wort, 2 µM) abolished the ability of Aβ to disperse Homer1b clusters (101.1±15.3%, cf. Aβ+Wort and Wort alone). Likewise, inhibition of the PI-3K downstream kinases PKB (with API-2, 30 µM) or mTor (with rapamycin, Rapa, 5 µM) prevented Aβ-induced declustering of Homer1b (90.6±8.4%, cf. Aβ+AP2 and API-2 alone; 95±8.2%, cf. Aβ+Rapa and rapamycin alone). Either the blockade of MEK/ERK pathway by pre-treatment with UO126 (10 µM; 70.9±4.8% UO126+Aβ vs UO126 alone) or cdk-5 inhibition by roscovitine (10 µM;63.7±5.8%, cf. Aβ+Rosco and Rosco alone) pre-treatment did affect Aβ-induced Homer declustering. (B) MEK/ERK pathway, but not PI-3K pathway, is required for Aβ-induced dispersal of Shank1. Pre-treatment with the PI-3K inhibitor wortmannin before Aβ treatment did not affect Aβ-induced Shank cluster dispersal (64.7±10.3%, cf. Wort+Aβ and Wort alone; p<0.05). Notably, wortmannin itself decreased Shank1 (but not Homer1b) cluster size (74.3±10.3%, cf. Wort and vehicle; p<0.05). Two structurally unrelated MEK inhibitors, UO126 (10 µM) and PD98059 (25 µM) prevented Aβ-induced declustering of Shank1 (96.8±12.1%, cf. PD98059+Aβ and PD98059 alone; 92.2±15.4%, cf. UO126+Aβ and UO126 alone). Likewise, the blockade of the Erk targeted kinase RSK by pre-treatment with SL0101-1 blocked Aβ effects (100.4±9.8%, p>0.05). Pre-treatment of neurons with the specific cdk-5 inhibitor roscovitine (Rosco, 10 µM) did not interfere with the ability of Aβ to reduce the size of Shank1 (72.6±10.8%, cf. Rosco+Aβ and Aβ alone) clusters. (C,D) PP2B is required for the dispersal of Homer1b, but not Shank1, clusters by Aβ. Pre-treatment with structurally unrelated PP2B inhibitors FK506 (10 µM) or cyclosporin (Cyclo, 100 µM) significantly blocked (p<0.05) Aβ-induced Homer1b declustering (98.8±10.4%, comparing FK506+Aβ vs. Aβ alone; 83.4±5.8%, comparing Aβ+Cyclo and Cyclo alone; and 61±7.9% in vehicle+Aβ-treated cells; *see panel C*); however, all PP2B inhibitors were ineffective in preventing Shank1 declustering after Aβ exposure (61.3±4.5%, cf. FK506+Aβ and FK506 alone; 90.8±12.4%, cf. Cyclo+Aβ and Cyclo alone, *see panel D*). For each condition, 8–12 neurons from 3–4 replicate experiments were considered (N = 8–12), and at least 50 synapses on each neuron were evaluated (on average, n = 500 puncta/condition).

Several signalling pathways downstream of NMDAR and VDCC may be potentially involved in Aβ-triggered remodelling of the PSD. Given the observation that new protein synthesis is required for Aβ-induced Homer declustering, we evaluated the importance of the PI-3K/mTOR pathway, a major regulator of protein synthesis. Inhibition of PI-3K signalling with wortmannin (2 µM) abolished the ability of Aβ to reduce synaptic levels of Homer1b (p<0.05; Fig. 6A), but not of Shank 1 (Fig. 6B); however, wortmannin alone caused significant shrinkage of Shank1 clusters (p<0.05; Fig. 6B) without affecting basal Homer1b cluster size (Fig. 6A). Blockade of the PI-3K downstream kinases, PKB and mTOR with API-2 (30 µM) and rapamycin (5 µM), respectively, also prevented the Aβ-induced reduction in synaptic Homer1b levels (p>0.05; Fig. 6A). However blockade of GSK-3β, another PI-3K target, failed to abrogate Aβ-induced shrinkage of Homer1b and Shank 1 clusters (data not shown).

To examine whether activation of the PI-3K/PKB pathway resulted from activation of NMDAR [36], VDCC [37] or both, we monitored the intensity of phospho-PKB immunostaining in neurons pre-treated with MK801, (10 µM), verapamil, (50 µM), or vehicle before treatment with soluble Aβ. Aβ triggered a significant increase in the phospho-PKB signal (143.2±17.1% *vs.* 100±14.2 in control, p<0.05; Supplementary Fig. S4). Whereas pre-treatment with MK801 significantly decreased phospho-PKB staining after Aβ application (77.1%±10.8, p<0.05; Supplementary Fig. S4A and B), verapamil did not significantly attenuate the Aβ-induced increase in PKB phosphorylation (139.1±11.1% of baseline, p<0.05; Aβ *vs.* Aβ+ verapamil, p>0.05). Together, these data indicate that PI-3K activation by Aβ is NMDAR-mediated.

Since the ERK pathway is known to regulate the interaction of Shank1 with the actin cytoskeleton [38] and ERK signalling ranks prominently among the signalling mechanisms activated by NMDAR, involvement of this pathway in the regulation of Shank1 and Homer1 by Aβ was examined. Two structurally unrelated MEK-ERK1/2 inhibitors, U0126 (10 µM) and PD98059 (25 µM), abrogated the effects of Aβ on the synaptic levels of Shank1 (p<0.05, Fig. 6B), but not of Homer1b (Fig. 6A) (data obtained with PD98059 are not shown). Confirming NMDAR as the origin of Aβ-triggered ERK activation, the significant increase in p-ERK levels following treatment with Aβ alone (184.6±47% of control, p<0.05) was abolished by MK801 pre-treatment (77±14.4 of control, Supplementary Fig. S5). Further, we found that RSK2/3, a kinase that acts downstream of ERK and is known to interact and directly phosphorylate Shank family proteins [38], was activated within 15 min of application of Aβ; the increase in phospho-RSK staining (180.2±24.5% from barely detectable levels under baseline conditions) was sensitive to NMDAR blockade with MK801 (114.0±11.8%) and UO126 (122.3±14.3%) (Supplementary Fig. S6). Finally, confirming the role of RSK in Aβ-induced Shank cluster dispersal, pretreatment of the cultures with the RSK inhibitor SL0101-1 (2 µM) prevented the dispersal of Shank clusters by Aβ (100.4±9.8%, p>0.05; Fig. 6B).

Protein phosphatase 2B (PP2B, calcineurin) signalling was recently identified as a mediator of Aβ-induced AMPAR endocytosis [39] and dendritic spine loss [4]. Consistent with a role of PP2B in Aβ-induced synaptic dysfunction, the PP2B inhibitors FK506 (10 µM) and cyclosporin (100 µM) abolished the Aβ-induced decrease in synaptic Homer1b (p<0.05; Fig. 6C), but not Shank (Fig. 6D), levels. The effects of Aβ on Homer1b could not be significantly abrogated by either okadaic acid (a general PP1/2A inhibitor, used at 2 µM) or cantharidine (a specific PP2A inhibitor, used at 0.2 µM) (not shown).

Lastly, the involvement of cdk5 (previously shown to mediate Aβ-induced degradation of PSD-95 [21], [40]) and of CaMKII and PKC α/γ (two Ca^2+^-dependent kinases implicated in synaptic physiology) in Aβ-induced Homer1b and Shank1 declustering were ruled out on the basis of results obtained with the cdk5 inhibitor roscovitine (10 µM) (Fig. 6A and 6B), KN-93 (5 µM) and Gö6893 (5 µM), respectively (Supplementary Fig. S7A and S7B).

### Aβ treatment reduces synaptic mGluR1

**Figure 7:**
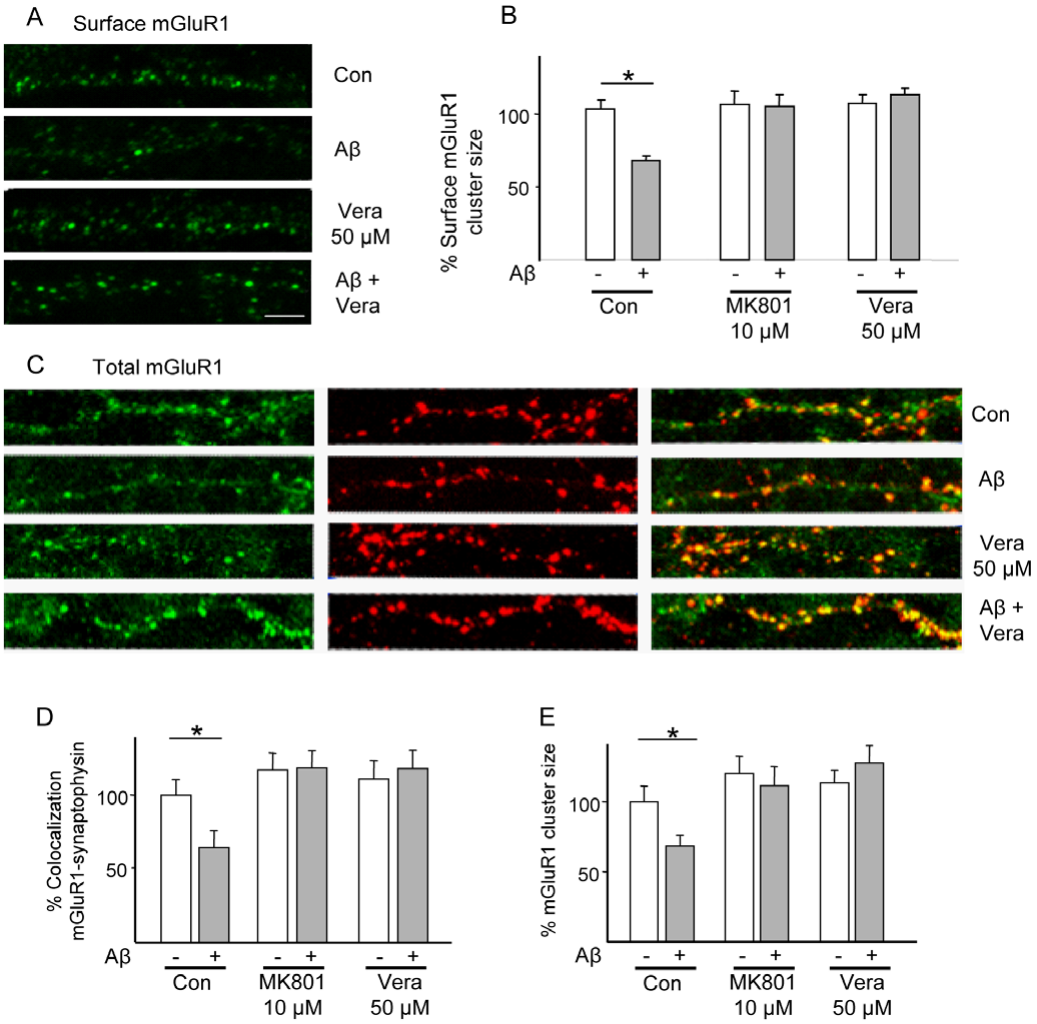
Dispersal of synaptic mGluR1 clusters by Aβ requires NMDAR and VDCC activity. Rat cortical neurons were treated with Aβ (1 µM) for 1 h (with or without co-treatment with the NMDAR antagonist MK801, or the VDCC blocker verapamil) and then immunostained for surface mGluR1 before fixation. (A, B) Aβ downregulates surface mGluR1 (surface cluster size 66.0±2.8%, N = 10, n = 300, p<0.05); pre-treatment with either MK801 (10 µM) or verapamil (50 µM) blocked this effect (102.9±10.5%, for MK801+Aβ vs. MK alone; 109.8±4.1%, for verapamil+Aβ vs. verapamil alone). (C, D) Aβ treatment results in decreased synaptic localization of total mGluR1 (ratio of mGluR1/synaptophysin immunopositive puncta, 47.3±5.7% of baseline, n = 600, p<0.05); pre-treatment with either MK801 or verapamil prevented this effect (102.0±7.2%, for MK801+Aβ vs. MK alone; 106.7±10.8% for verapamil+Aβ vs. verapamil alone). (C, E) Aβ treatment results in reduced size of total synaptic mGluR1 clusters (68.1±8.0%, N = 10, n = 600, p<0.05); pre-treatment with either MK801 or verapamil abolished Aβ-induced cluster shrinkage (93±11.1%, for MK801+Aβ vs. MK alone; 112.4±10.7%, for verapamil+Aβ vs. verapamil alone). Scale bar represents 5 µm.

Since mGluR1 trafficking to the surface relies on interactions with Homer1b [17], [41], it was of interest to examine the consequences of Aβ-induced decreases in synaptic levels of Homer1b on mGluR1 localization. Immunostaining revealed a major decrease in surface mGluR1 cluster size after Aβ treatment (surface cluster size 66.0±2.8%, n = 10, p<0.05, Fig. 7A and 7B), an effect that was prevented by treatment with either MK801 (10 µM) or verapamil (50 µM). mGluR1 is regulated by constitutive endocytosis, a process that is subject to retardation upon binding of Homer proteins to mGluR1 [41]–[43]. Here, the size of mGluR1 synaptic clusters was evaluated to examine whether the Aβ-induced loss of surface mGluR1 resulted from endocytosis alone or whether it also involved decreases in overall synaptic mGluR1 content. Under baseline conditions, mGluR1 immunoreactivity was predominantly found in the perikaryon and dendrites of neurons [17] in approximately 60% of synaptophysin-positive puncta (Fig. 7C and 7D). The number of mGluR1-positive synapses was markedly decreased within 1 h of Aβ application (47.3±5.7% of control, n = 10; p<0.05; Fig. 7C and 7D). Likewise, the size of synaptic mGluR1 clusters was decreased after exposure to Aβ (65±6.5% of control; p<0.05; Fig. 7C and 7E). The effects of Aβ on synaptic mGluR1 localization (Fig. 7C and 7D) and cluster size (Fig. 7E) proved to be sensitive to MK801 (10 µM) and verapamil (50 µM), indicating their mediation by NMDAR and VDCC, respectively (Fig. 7C and 7D).

## Discussion

Oligomeric Aβ has been implicated in the pathogenesis of Alzheimer's disease [2], [8] but a physiological role for Aβ in the regulation of synaptic homeostasis has also been hypothesized [44]. Several lines of evidence suggest that soluble Aβ may contribute to the early stages of AD by impairing synaptic physiology: (i) soluble Aβ levels correlate strongly with loss of synapses as well as with severity of cognitive impairment in AD patients [6] and in transgenic mouse models [7], [45], (ii) Aβ oligomers acutely impair synaptic plasticity and cognitive functions [3], [7], [8], and (iii) Aβ oligomers influence the overall levels, localization and trafficking of several receptors (including NMDAR and AMPAR) and signalling molecules [39], [46], [47].

In the present investigations, Aβ was used at a dose (1 µM) and over a period of time (1 h), conditions that do not favor its aggregation into fibrils [24]–[26]. Our data identify the remodelling of the PSD and the dispersal of scaffold proteins clustered in the post-synaptic site as early events triggered by Aβ oligomers. We show that Aβ rapidly disrupts the ultrastructural integrity and thickness of the PSD, inducing morphological changes that are reminiscent of the phenotype found in mice in which selected PSD constituents, including Shank [30], are knocked out. Extending previous findings that Aβ treatment leads to a loss of PSD-95 [21], [22], we here found this treatment to lead to a significant decrease in the size of synaptic clusters (loss) of two other PSD proteins, Homer1b and Shank1.

Interestingly, our investigations show that distinct mechanisms underlie Aβ-triggered loss of synaptic Homer1b and Shank1. In contrast to previous demonstrations that the loss of synaptic PSD-95 requires proteasomal activity [21], [33], Shank1 loss was found to occur independently of proteasomal activity over the timeframe monitored in the present study, although the possibility of degradation of the protein over longer periods cannot be ruled out [cf.33]. Remarkably, a different mechanism, namely *de novo* protein synthesis, was found to underlie the loss of synaptic Homer1b after application of Aβ. Since the reduction in synaptic Homer1b clusters was not accompanied by a change in whole-cell levels of the protein (or its truncated forms), it would appear that Aβ treatment leads to cluster dissociation and relocation to another cellular compartment [cf.34]. The presented data on Shank1 and Homer1b, together with previous findings for PSD-95 [21], show that Aβ can selectively employ either protein synthesis or degradation to disrupt the PSD; our results complement other work on a different model of synaptic plasticity [48].

Declustering of Homer and Shank, like that of PSD-95 [21], was found to require NMDAR activity (and agonist-induced activation of NMDAR alone was sufficient to mimic the Aβ effect). Therefore, the NMDAR is a major hub for the regulation of PSD structure and composition and its activation appears to be essential for triggering disassembly of the PSD; however, our study demonstrates that divergent downstream signalling pathways regulate the demise of each PSD component. Specifically, we found that Aβ-induced Homer declustering requires activated VDCC as an additional source of Ca^++^ and other signalling molecules; importantly, VDCC activation alone however did not mimic the actions of Aβ. These observations are consistent with those of Okabe et al. [34] who reported that NMDAR- and VDCC-regulated Ca^2+^ spikes promote Homer1b clustering whereas Ca^2+^ levels that rise and reach a plateau result in the shrinkage of Homer1b clusters.

The PI-3K/mTor signalling pathway, acting downstream of NMDAR, was identified as an important mediator of Aβ-induced Homer1b cluster dispersal (see Supplementary Fig. S8). Since PI-3K/mTOR is a master regulator of protein synthesis [49] it is plausible that this pathway links NMDAR activation to the synthesis of protein(s) ultimately involved in Homer cluster disassembly. Consistent with a previous demonstration that NMDAR-dependent activation of PI-3K requires the binding to a phospho-tyrosine residue on the cytoplasmic tail of the NR2 subunit of the NMDAR [50], inhibition of the src tyrosine kinase in the present study interfered with Homer1b declustering. A role for protein phosphatase 2B (PPB2), acting downstream of NMDAR [51], in the control of Homer1b cluster disassembly was also demonstrated in the present work. The recent implication of PPB2 and its downstream target cofillin in Aβ-induced loss of dendritic spines [4] suggests that this molecule may serve as a mechanistic link between Homer1b cluster dispersal and dendritic spine loss [4], [12].

Signalling pathways that are distinct from those involved in the regulation of Homer1b declustering were found to mediate NMDAR-triggered dispersal of Shank1 clusters. While roles for the PI-3K and PP2B pathways were dismissed, we demonstrated serial activation of ERK and RSK upon NMDAR activation. Previous work demonstrated that ERK/RSK activation leads to the phosphorylation of Shank protein and a reduction in its affinity for the actin-binding protein, cortactin [38]. Since Shank clustering depends upon actin dynamics [52], the probability of Shank declustering after interference with the Shank-cortactin interaction through the ERK-RSK pathway is high.

Homer1b is known to critically affect the clustering and trafficking of mGluR1 as well as its delivery to the cell surface from where mGluR1 is removed by constitutive and regulated internalisation and degradation [17], [41]–[43]. The present finding that Aβ causes a significant decrease in the surface expression of mGluR1 implies endocytosis of the receptor. Further, our studies show that the loss of surface mGluR1 can be prevented by blockade of either NMDAR or VDCC, paralleling our observations with respect to Homer1b declustering; it may therefore be inferred that loss of synaptic Homer1b is a key event in the downregulation of surface levels of mGluR1. Interestingly, Aβ induces a decrease in the total synaptic content of mGluR1; this finding suggests receptor endocytosis and degradation rather than receptor recycling. In brief, while the loss of Homer1b and mGluR1 is likely to contribute to Aβ-triggered demise of the PSD, our results are compatible with the view that mGluR1 surface clustering is tightly coupled to mGluR1 interactions with Homer1b [41]. Thus, Homer1b declustering is directly involved in the internalisation of mGluR1. Accordingly, we suggest that loss of synaptic Homer1b and mGluR1 mediates a major part of the impact of Aβ on synaptic physiology [cf. 53]–[55].

In summary, our results suggest that soluble Aβ affects the composition and overall structure of the PSD by triggering its disassembly and, ultimately, synaptic loss. The results presented here and previously [21] highlight the remarkable diversity of signaling mechanisms through which Aβ regulates the synaptic levels of three strongly interacting scaffold proteins in the mature PSD. Despite its structural complexity, the PSD may be viewed as a “flexible matrix” undergoing continuous turnover and structural plasticity [52], [56]. In this context, the relative abundance and binding affinity of each component could be regulated independently [cf. 57], [58], ultimately leading to either changes in PSD composition or its complete disassembly.

## Supporting Information

Figure S1 Ab effect on Homer1b and Shank1 clusters are dose-dependent. (A,B) Cultured rat fronto-cortical neurons were treated with Aβ at doses ranging from 100 pM to 1 µM for 1 h; thereafter they were immunostained for Homer1b or Shank (significant differences are indicated by asterisks, p<0.05).
(4.38 MB TIF)

Figure S2 Homer1b and Shank1 cluster dispersal by Aβ does not depend upon AMPAR or mGluR activity. (A, B) Cultured fronto-cortical neurons were pre-treated with the AMPAR blocker NQBX (50 µM, 45 min) or the mGluR I/II blocker E4CPG (10 µM) before exposure to Aβ (1 µM, 1 h), after which they were fixed and immunostained for synaptophysin and either Homer1b (panel A) or Shank1 (panel B). Neither NBQX nor E4CPG interfered with the ability of Aβ to reduce Homer1b (66±4.3%, cf. NQBX+Aβ and NQBX alone, p<0.05) or Shank1 (62±4.9%, cf. NQBX+Aβ and NQBX alone, p<0.05; 69.3±4.4%, cf. E4CPG+Aβ and E4CPG alone, p<0.05; 59.2±2.7%, cf. E4CPG+Aβ vs. E4CPG alone, p<0.05) cluster sizes.
(4.27 MB TIF)

Figure S3 L-type, but not T-type, calcium channels are required for Aβ-induced dispersal of Homer1b clusters. Neurons were pre-treated with the L-type VDCC blocker nifedipine (40 µM, 45 min) or with the T-type blocker NiCl_2_ (400 µM, 45 min) before being treated with Aβ (1 µM, 1 h). Consistent with the results obtained with the structurally unrelated VDCC blocker verapamil (see Fig. 4), nifedipine effectively prevented Homer1b cluster dispersal by Aβ (95.8±8.4%, cf. nifedipine+Aβ and nifedipine alone, p>0.05), whereas NiCl_2_ proved ineffective in blocking the actions of Aβ (64.7±5.2%, cf. Aβ+NiCl_2_ and NiCl_2_ alone, p<0.05).
(1.97 MB TIF)

Figure S4 Aβ activates the PKB pathway through NMDAR. Rat cortical neurons were starved (see Methods) for 2 h before pre-treatment (45 min) with the NMDAR antagonist MK801 (10 µM), the VDCC blocker verapamil (50 µM) or vehicle before exposure to Aβ (1 µM, 10 min). Cells were fixed and immunostained for p-PKB (Thr308) and synaptophysin. (A) Representative images of p-PKB-immunostained cells (column 1: intensity-coded image, column 2: actual palette), synaptophysin-stained cells (column 3), and the resulting overlay (column 4) are shown. Cells included in the evaluation expressed cytoplasmic synaptophysin as well as punctate synaptophysin immunoreactivity along their processes. (B) shows p-PKB immunofluorescence intensity (after background subtraction). Aβ treatment triggered an increase in p-PKB fluorescence intensity (143.2±17.1% of baseline, n = 50, p<0.05). Pre-treatment with MK801 abrogated Aβ-induced increases in p-PKB immunoreactivity (77.1%±10.8% of baseline, n = 30), whereas pre-treatment with verapamil had only a minor effect on p-PKB immunofluorescence (139.1±11.1% of baseline, p<0.05). Scale bar represents 10 µM.
(8.06 MB TIF)

Figure S5 Aβ activates ERK phosphorylation through NMDAR. Fronto-cortical neurons were starved (see Methods) for 2 h before pre-treatment (45 min) with the NMDAR antagonist MK801 (10 µM) or vehicle before exposure to Aβ (1 µM, 10 min). Cells were fixed and immunostained for p-ERK (Thr202/Tyr204) and synaptophysin. (A) shows representative images of p-ERK immunostaining (column 1: intensity-coded image; column 2: actual palette), synaptophysin immunostaining (column 3) and the resulting overlay (column 4). Cells included in the evaluation expressed cytoplasmic synaptophysin as well as punctate synaptophysin immunoreactivity along their processes. (B) demonstrates p-ERK immuno-fluorescence intensity (after background subtraction). Aβ treatment led to an increase in p-ERK fluorescence intensity (184.6±47% of baseline, n = 50, p<0.05). Pre-treatment with MK801 abrogated Aβ-induced p-ERK immunoreactivity (77±14.4% of baseline, n = 30). Scale bar represents 10 µM.
(9.46 MB TIF)

Figure S6 Aβ activates RSK phosphorylation through NMDAR and Erk pathway. Fronto-cortical neurons were starved (see Methods) for 2 h before pre-treatment (45 min) with the NMDAR antagonist MK801 (10 µM), the MEK inhibitor UO126 (10 µM) or vehicle before exposure to Aβ (1 µM, 15 min). Cells were fixed and immunostained for p-RSK and synaptophysin. (A) shows representative images of p-RSK immunostaining (column 1: intensity-coded image; column 2: actual palette), synaptophysin immunostaining (column 3) and the resulting overlay (column 4). Cells included in the evaluation expressed cytoplasmic synaptophysin as well as punctate synaptophysin immunoreactivity along their processes. (B) demonstrates p-ERK immuno-fluorescence intensity (after background subtraction). Aβ treatment led to an increase in p-ERK fluorescence intensity (180.2±24.5 of baseline, n = 50, p<0.05). Pre-treatment with MK801 and UO126 largely abrogated Aβ-induced p-RSK immunoreactivity (114.3±11.8% and 122.3±14.3% of baseline respectively, n = 30). Scale bar represents 10 µM.
(8.14 MB TIF)

Figure S7 Differential kinase requirements in the dispersal of Homer1b and Shank1 clusters by Aβ. Cortical neurons were pre-treated with KN93 (5 µM) or Gö6893 (5 µM) for 45 min before addition of Aβ (1 µM, 1 h). (A, B) demonstrate that CaMKII and PKCα/γ are not required for manifestation of the ability of Aβ to induce dispersal of Homer1b and Shank1 clusters. Neither KN93 nor Gö6893 were effective at blocking the effects of Aβ on Homer1b (63.9±9.4% for KN93+Aβ vs. KN93 alone, p<0.05; and 68.4±4.4% for Gö6893+Aβ vs. Gö6893 alone, p<0.05). Similarly, neither inhibitor influenced the actions of Aβ on Shank1 clusters (64.4±6.8% for KN93+Aβ vs. KN93 alone, p<0.05; and 73.6±8% for Gö6893+Aβ vs. Gö6893 alone, p<0.05). Notably, Gö6893 itself (but not KN93) led to a marked decrease in Shank1 cluster size (66.5±10.9%, cf. Gö6893 and control, p<0.05).
(4.43 MB TIF)

Figure S8 Divergent signalling pathways mediate Aβ effects on Homer1b, Shank1 and PSD-95 and regulate Aβ-induced PSD remodelling. Aβ-triggered signalling pathways leading to PSD disruption are shown. Homer cluster disassembly relies on PI-3K/mTot pathway and PP2B pathway activity, whereas Shank cluster disassembly only requires ERK/RSK pathway activity (present work). In contrast, PSD-95 loss involves the activity of cdk-5. For each pathway, the inhibitors used in the paper are shown.
(7.38 MB TIF)
