# Supplementary figures and images for: Disassembly of Shank and Homer Synaptic Clusters Is Driven by Soluble β-Amyloid1-40 through Divergent NMDAR-Dependent Signalling Pathways
Source: PLoS One. 2009 Jun 23;4(6):e6011. doi: 10.1371/journal.pone.0006011 (PMC2695780; doi:10.1371/journal.pone.0006011)

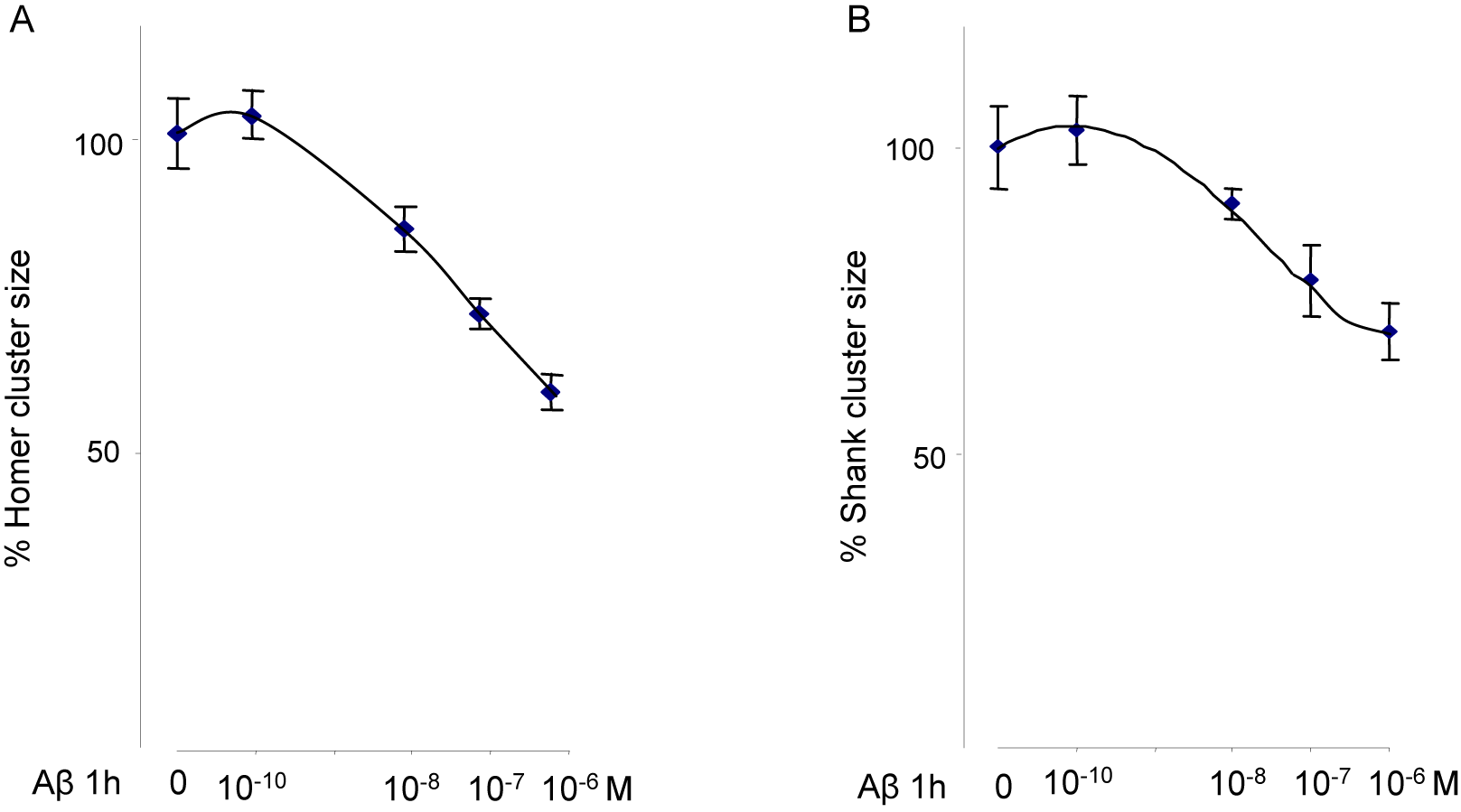

Supplement: Figure S1 — Ab effect on Homer1b and Shank1 clusters are dose-dependent. (A,B) Cultured rat fronto-cortical neurons were treated with Aβ at doses ranging from 100 pM to 1 µM for 1 h; thereafter they were immunostained for Homer1b or Shank (significant differences are indicated by asterisks, p<0.05). (4.38 MB TIF) [file pone.0006011.s001.tif]

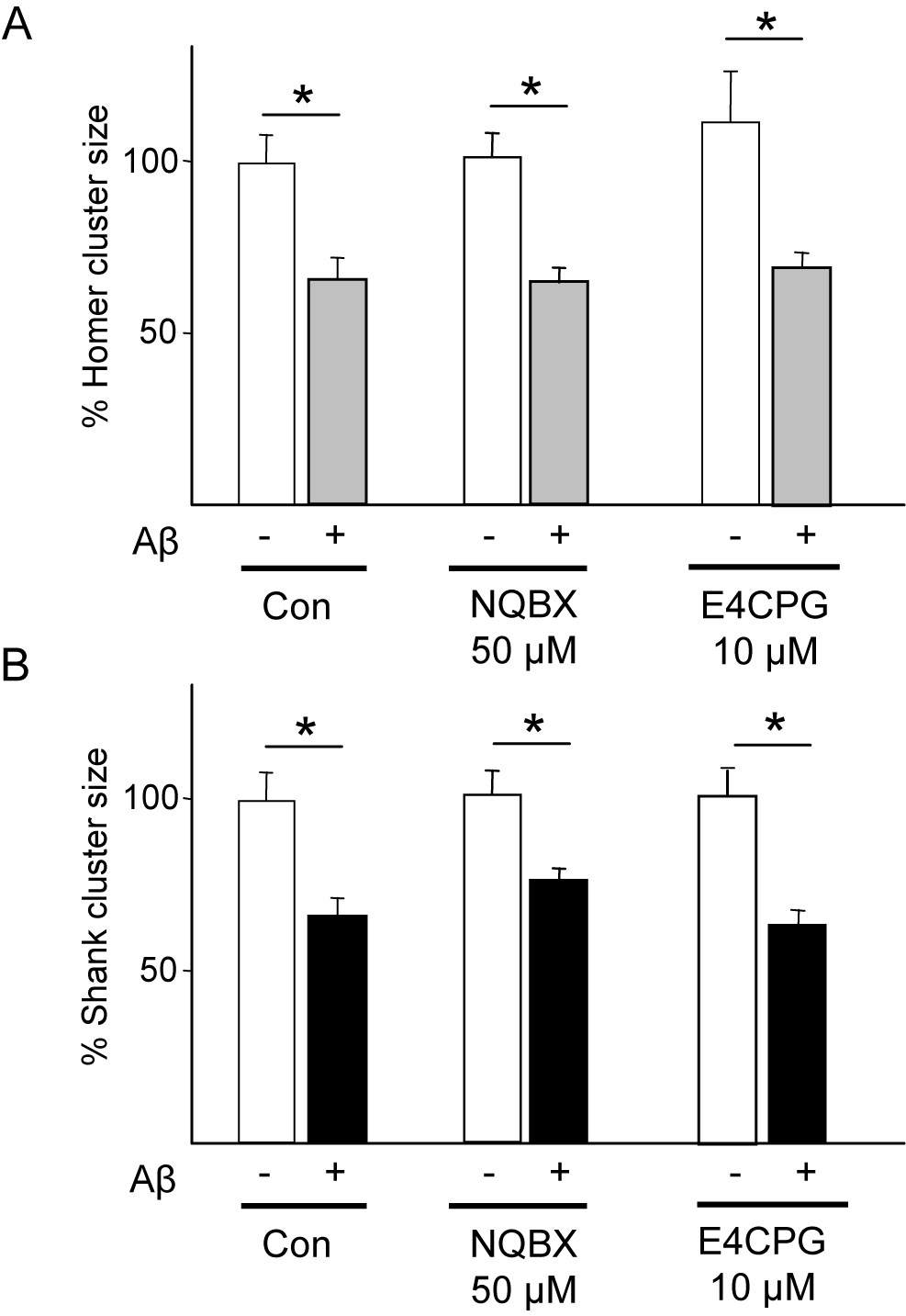

Supplement: Figure S2 — Homer1b and Shank1 cluster dispersal by Aβ does not depend upon AMPAR or mGluR activity. (A, B) Cultured fronto-cortical neurons were pre-treated with the AMPAR blocker NQBX (50 µM, 45 min) or the mGluR I/II blocker E4CPG (10 µM) before exposure to Aβ (1 µM, 1 h), after which they were fixed and immunostained for synaptophysin and either Homer1b (panel A) or Shank1 (panel B). Neither NBQX nor E4CPG interfered with the ability of Aβ to reduce Homer1b (66±4.3%, cf. NQBX+Aβ and NQBX alone, p<0.05) or Shank1 (62±4.9%, cf. NQBX+Aβ and NQBX alone, p<0.05; 69.3±4.4%, cf. E4CPG+Aβ and E4CPG alone, p<0.05; 59.2±2.7%, cf. E4CPG+Aβ vs. E4CPG alone, p<0.05) cluster sizes. (4.27 MB TIF) [file pone.0006011.s002.tif]

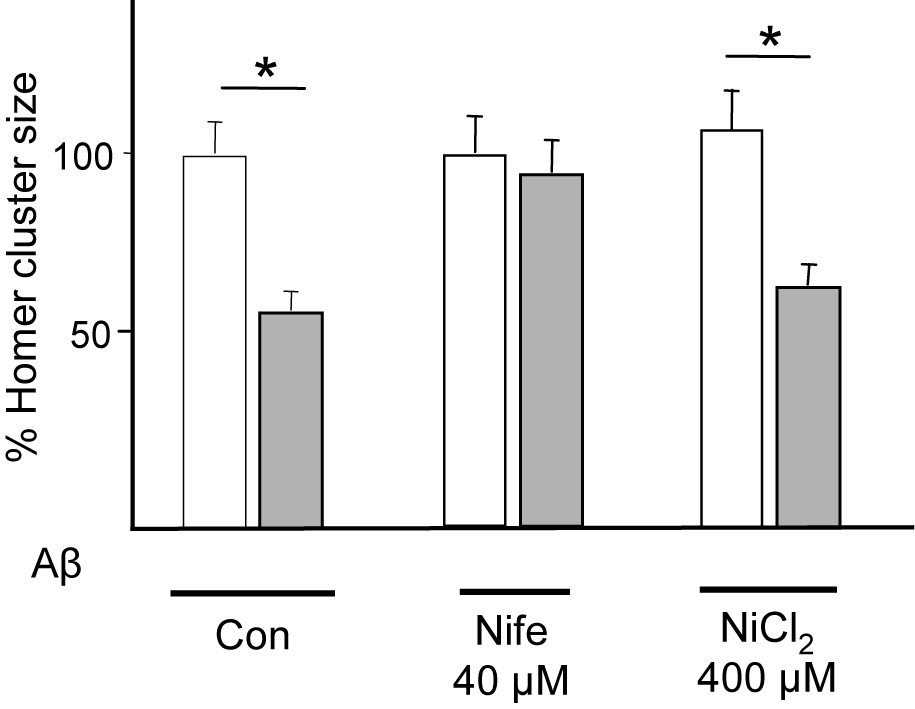

Supplement: Figure S3 — L-type, but not T-type, calcium channels are required for Aβ-induced dispersal of Homer1b clusters. Neurons were pre-treated with the L-type VDCC blocker nifedipine (40 µM, 45 min) or with the T-type blocker NiCl2 (400 µM, 45 min) before being treated with Aβ (1 µM, 1 h). Consistent with the results obtained with the structurally unrelated VDCC blocker verapamil (see Fig. 4), nifedipine effectively prevented Homer1b cluster dispersal by Aβ (95.8±8.4%, cf. nifedipine+Aβ and nifedipine alone, p>0.05), whereas NiCl2 proved ineffective in blocking the actions of Aβ (64.7±5.2%, cf. Aβ+NiCl2 and NiCl2 alone, p<0.05). (1.97 MB TIF) [file pone.0006011.s003.tif]

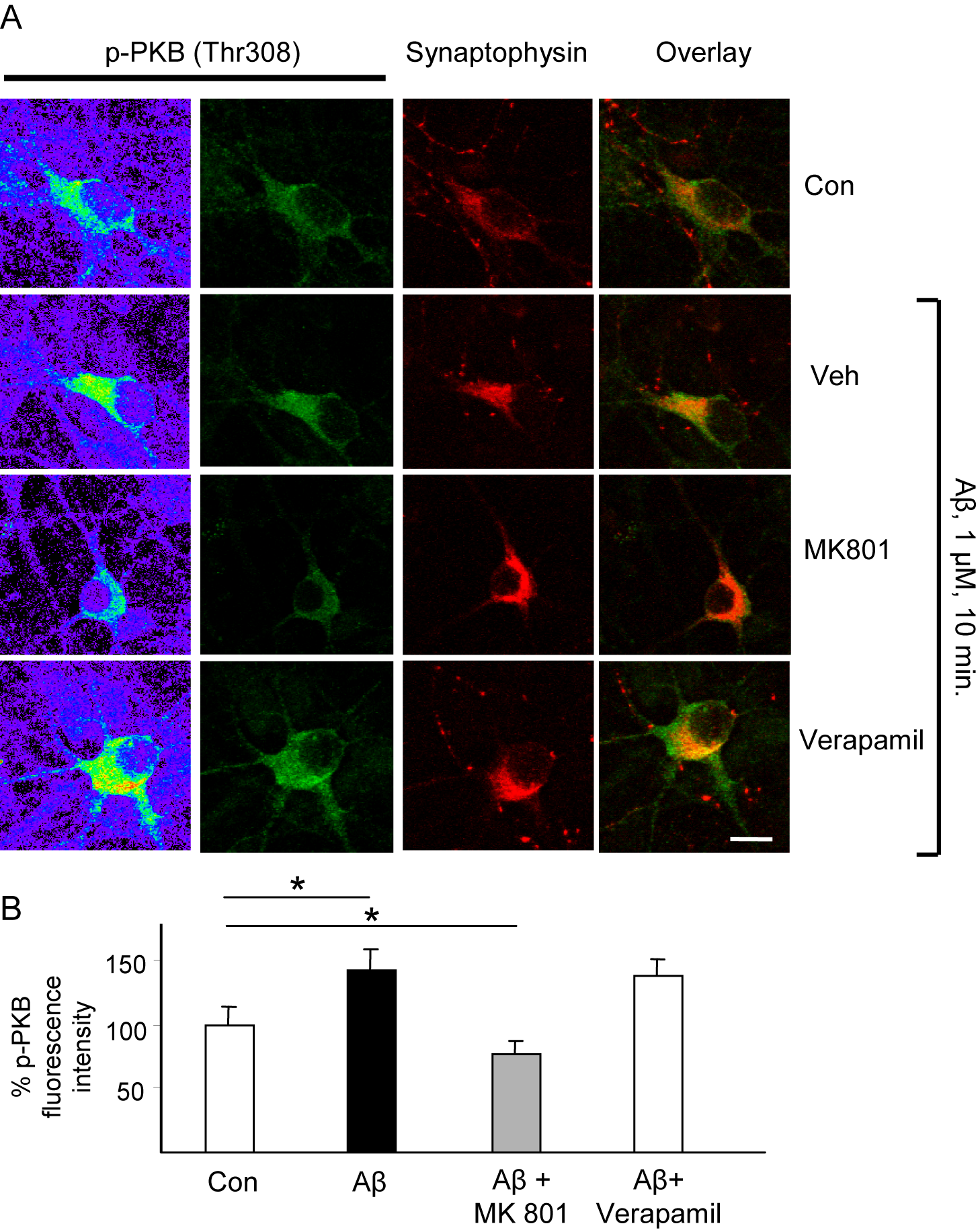

Supplement: Figure S4 — Aβ activates the PKB pathway through NMDAR. Rat cortical neurons were starved (see Methods) for 2 h before pre-treatment (45 min) with the NMDAR antagonist MK801 (10 µM), the VDCC blocker verapamil (50 µM) or vehicle before exposure to Aβ (1 µM, 10 min). Cells were fixed and immunostained for p-PKB (Thr308) and synaptophysin. (A) Representative images of p-PKB-immunostained cells (column 1: intensity-coded image, column 2: actual palette), synaptophysin-stained cells (column 3), and the resulting overlay (column 4) are shown. Cells included in the evaluation expressed cytoplasmic synaptophysin as well as punctate synaptophysin immunoreactivity along their processes. (B) shows p-PKB immunofluorescence intensity (after background subtraction). Aβ treatment triggered an increase in p-PKB fluorescence intensity (143.2±17.1% of baseline, n = 50, p<0.05). Pre-treatment with MK801 abrogated Aβ-induced increases in p-PKB immunoreactivity (77.1%±10.8% of baseline, n = 30), whereas pre-treatment with verapamil had only a minor effect on p-PKB immunofluorescence (139.1±11.1% of baseline, p<0.05). Scale bar represents 10 µM. (8.06 MB TIF) [file pone.0006011.s004.tif]

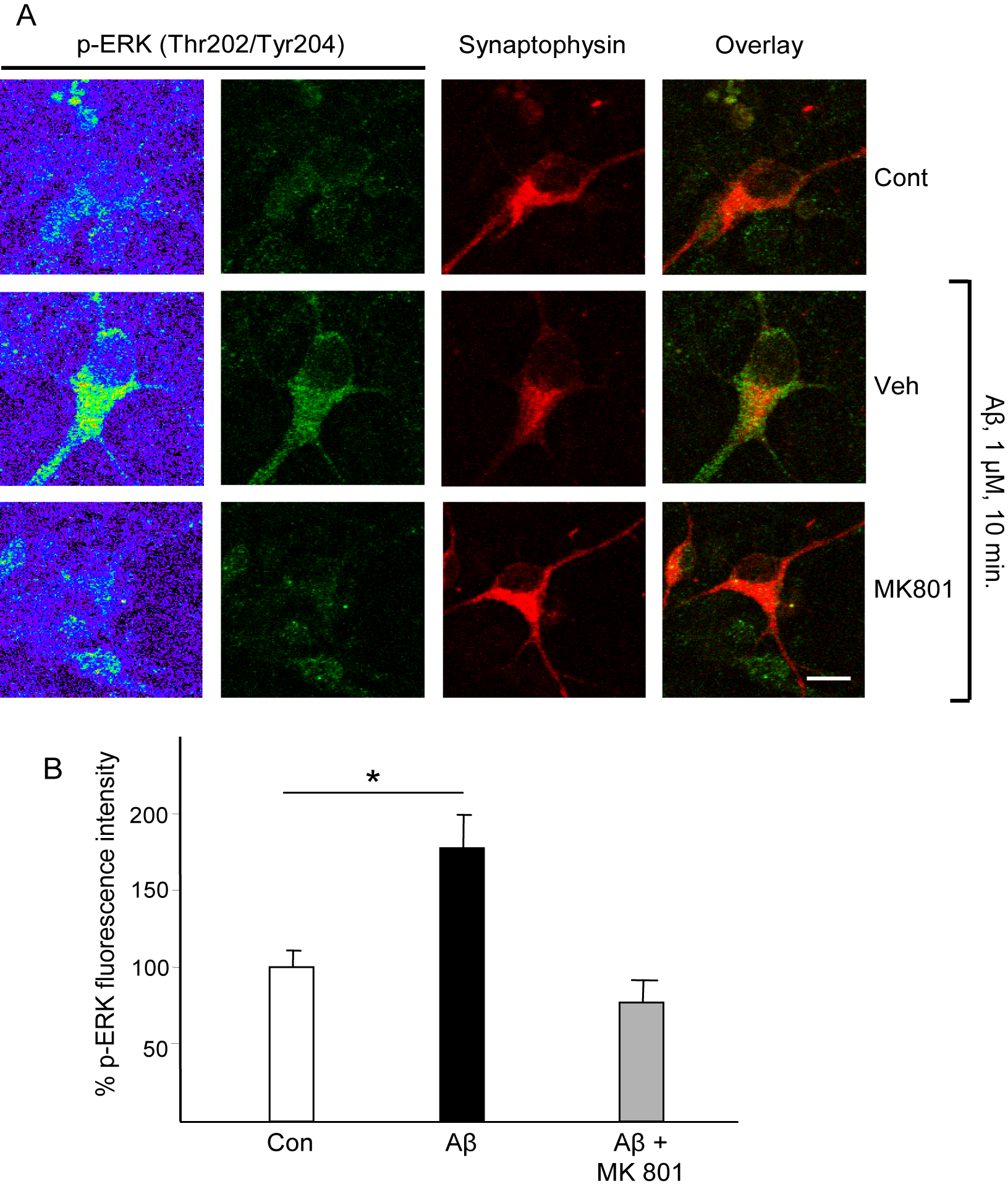

Supplement: Figure S5 — Aβ activates ERK phosphorylation through NMDAR. Fronto-cortical neurons were starved (see Methods) for 2 h before pre-treatment (45 min) with the NMDAR antagonist MK801 (10 µM) or vehicle before exposure to Aβ (1 µM, 10 min). Cells were fixed and immunostained for p-ERK (Thr202/Tyr204) and synaptophysin. (A) shows representative images of p-ERK immunostaining (column 1: intensity-coded image; column 2: actual palette), synaptophysin immunostaining (column 3) and the resulting overlay (column 4). Cells included in the evaluation expressed cytoplasmic synaptophysin as well as punctate synaptophysin immunoreactivity along their processes. (B) demonstrates p-ERK immuno-fluorescence intensity (after background subtraction). Aβ treatment led to an increase in p-ERK fluorescence intensity (184.6±47% of baseline, n = 50, p<0.05). Pre-treatment with MK801 abrogated Aβ-induced p-ERK immunoreactivity (77±14.4% of baseline, n = 30). Scale bar represents 10 µM. (9.46 MB TIF) [file pone.0006011.s005.tif]

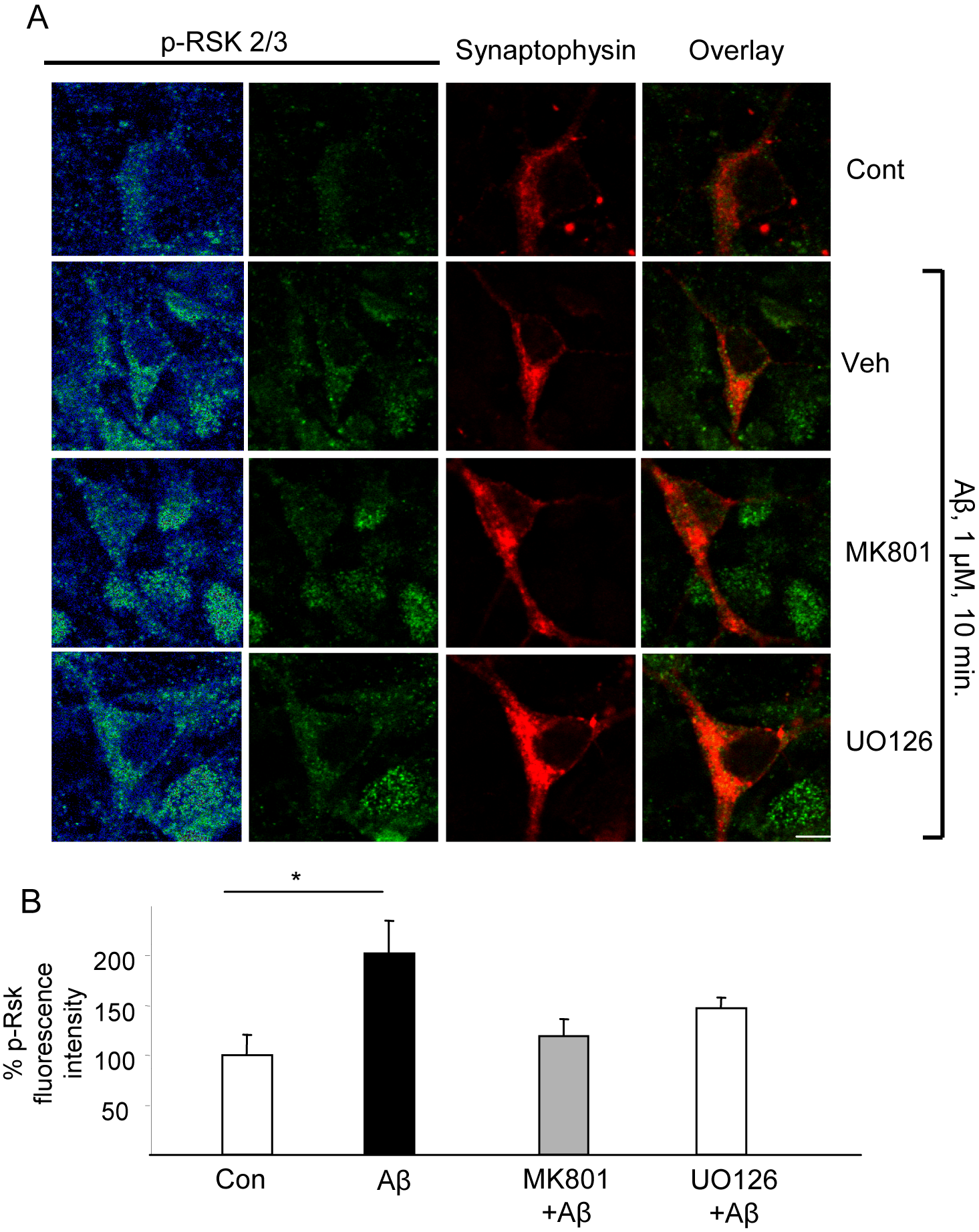

Supplement: Figure S6 — Aβ activates RSK phosphorylation through NMDAR and Erk pathway. Fronto-cortical neurons were starved (see Methods) for 2 h before pre-treatment (45 min) with the NMDAR antagonist MK801 (10 µM), the MEK inhibitor UO126 (10 µM) or vehicle before exposure to Aβ (1 µM, 15 min). Cells were fixed and immunostained for p-RSK and synaptophysin. (A) shows representative images of p-RSK immunostaining (column 1: intensity-coded image; column 2: actual palette), synaptophysin immunostaining (column 3) and the resulting overlay (column 4). Cells included in the evaluation expressed cytoplasmic synaptophysin as well as punctate synaptophysin immunoreactivity along their processes. (B) demonstrates p-ERK immuno-fluorescence intensity (after background subtraction). Aβ treatment led to an increase in p-ERK fluorescence intensity (180.2±24.5 of baseline, n = 50, p<0.05). Pre-treatment with MK801 and UO126 largely abrogated Aβ-induced p-RSK immunoreactivity (114.3±11.8% and 122.3±14.3% of baseline respectively, n = 30). Scale bar represents 10 µM. (8.14 MB TIF) [file pone.0006011.s006.tif]

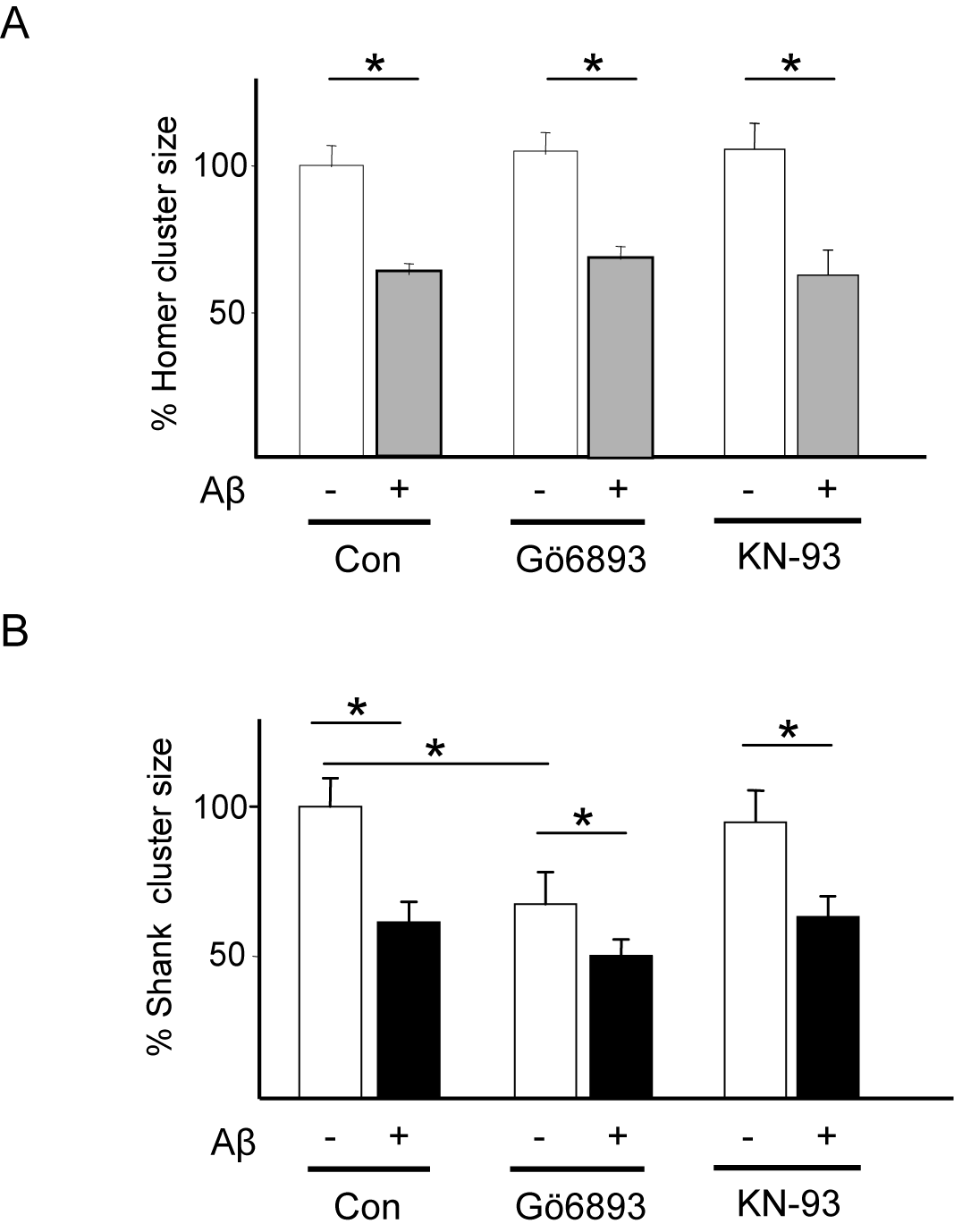

Supplement: Figure S7 — Differential kinase requirements in the dispersal of Homer1b and Shank1 clusters by Aβ. Cortical neurons were pre-treated with KN93 (5 µM) or Gö6893 (5 µM) for 45 min before addition of Aβ (1 µM, 1 h). (A, B) demonstrate that CaMKII and PKCα/γ are not required for manifestation of the ability of Aβ to induce dispersal of Homer1b and Shank1 clusters. Neither KN93 nor Gö6893 were effective at blocking the effects of Aβ on Homer1b (63.9±9.4% for KN93+Aβ vs. KN93 alone, p<0.05; and 68.4±4.4% for Gö6893+Aβ vs. Gö6893 alone, p<0.05). Similarly, neither inhibitor influenced the actions of Aβ on Shank1 clusters (64.4±6.8% for KN93+Aβ vs. KN93 alone, p<0.05; and 73.6±8% for Gö6893+Aβ vs. Gö6893 alone, p<0.05). Notably, Gö6893 itself (but not KN93) led to a marked decrease in Shank1 cluster size (66.5±10.9%, cf. Gö6893 and control, p<0.05). (4.43 MB TIF) [file pone.0006011.s007.tif]

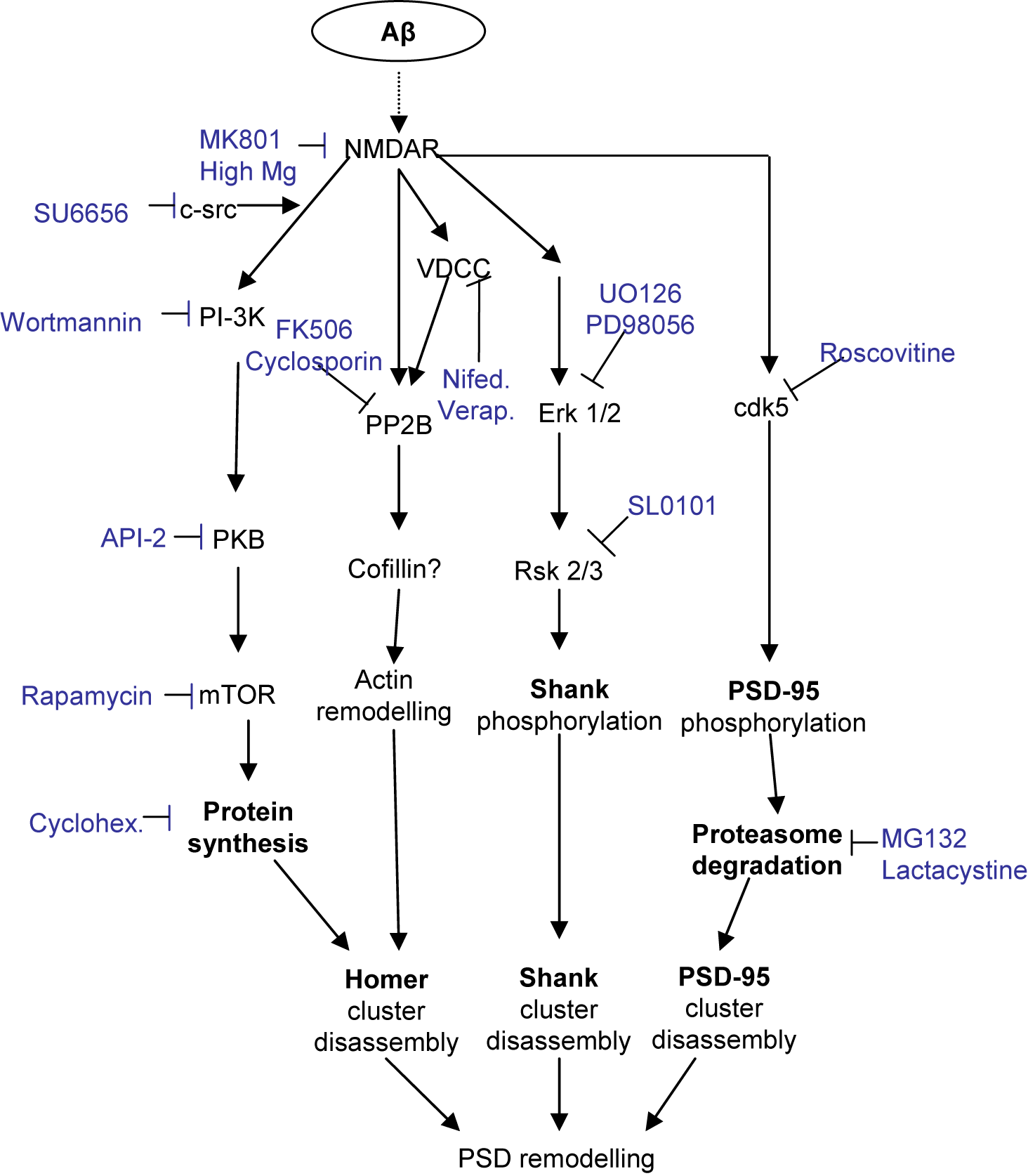

Supplement: Figure S8 — Divergent signalling pathways mediate Aβ effects on Homer1b, Shank1 and PSD-95 and regulate Aβ-induced PSD remodelling. Aβ-triggered signalling pathways leading to PSD disruption are shown. Homer cluster disassembly relies on PI-3K/mTot pathway and PP2B pathway activity, whereas Shank cluster disassembly only requires ERK/RSK pathway activity (present work). In contrast, PSD-95 loss involves the activity of cdk-5. For each pathway, the inhibitors used in the paper are shown. (7.38 MB TIF) [file pone.0006011.s008.tif]
